# Substrate temperature modulated optical characterizations of α-CdIn_2_Se_4_ thin films grown by pulsed laser deposition technique

**DOI:** 10.1039/d5ra03888j

**Published:** 2025-07-23

**Authors:** S. D. Dhruv, Tanvi Dudharejiya, Sergei A. Sharko, Aleksandra I. Serokurova, Nikolai N. Novitskii, D. L. Goroshko, Parth Rayani, Jagruti Jangale, Vanaraj Solanki, P. B. Patel, U. B. Trivedi, J. H. Markna, Bharat Kataria, D. K. Dhruv

**Affiliations:** a Natubhai V. Patel College of Pure and Applied Sciences, The Charutar Vidya Mandal (CVM) University Vallabh Vidyanagar-388120 Anand Gujarat India dhananjaydhruv@rediffmail.com; b Department of Nanoscience and Advanced Materials, Saurashtra University Rajkot-360005 Gujarat India; c Laboratory of Magnetic Films Physics, Scientific-Practical Materials Research Centre of National Academy of Sciences of Belarus 220072 Minsk Belarus; d Institute of Automation and Control Processes Far Eastern Branch of the Russian Academy of Sciences 5 Radio St. Vladivostok 690041 Russia; e Government Science College, Maharaja Krishnakumarsinhji Bhavnagar University Gariyadhar-364505 Bhavnagar Gujarat India; f Dr K.C. Patel R & D Centre, Charotar University of Science and Technology Changa-388421 Gujarat India; g Department of Electronics, Sardar Patel University Vallabh Vidyanagar-388120 Anand Gujarat India

## Abstract

The current study examines the effect of substrate temperature (*T*_s_) on the optical characteristics of CdIn_2_Se_4_ thin films grown by the pulsed laser deposition technique using a UV-Vis-NIR spectrophotometer. Transmittance maxima of CdIn_2_Se_4_ thin films shift with a change in *T*_s_, exhibit high absorption in the visible region, and depict an absorption coefficient (*α*) of ≃10^7^ m^−1^. Refractive index spectra of CdIn_2_Se_4_ thin films controlled by *T*_s_ reflect crests at characteristic wavelengths (*λ*_c_) and a wavelength (*λ*) higher than *λ*_c_; spectra display normal dispersion. The extinction coefficient (*k*) of *T*_s_ tempered CdIn_2_Se_4_ thin films decreases as *λ* increases and reaches a minimum at *λ*_c_; the contribution of free carrier absorption can explain the increase in *k* values after *λ*_c_. The effect of *T*_s_ on the optical band gap energy (*E*_g_) of the CdIn_2_Se_4_ thin films is discussed. For *T*_s_-modulated CdIn_2_Se_4_ thin films, dielectric constants, loss tangent, Urbach energy, Urbach absorption coefficient, and optical and electrical conductivities have been inferred. Peak values of the volume and surface energy loss functions of CdIn_2_Se_4_ thin films were retrieved. A Fourier transform infrared spectrophotometer verified the purity of CdIn_2_Se_4_ thin films formed at different *T*_s_. The current study indicates that CdIn_2_Se_4_ thin films are promising options for designing and developing upcoming high-efficiency opto-electronic devices. Implications are discussed.

## Abstract 2

By capturing the FTIR spectra of substrate temperature modulated CdIn_2_Se_4_ thin films, the purity of the films generated at various substrate temperatures was confirmed.

## Graphical abstract

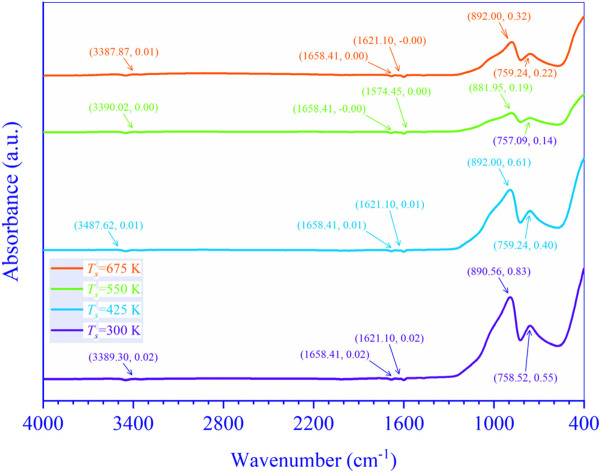

## 1 Introduction

Due to their potential uses in thermo-electric materials,^1^ semiconductor instrumentation,^2^ switching devices,^3,4^ photocatalysts,^5–7^ photoconductors,^8,9^ non-linear optical devices,^10,11^ optical filters,^12^ optoelectronics,^13^ solar cells,^14–17^ and more, ternary semiconducting compounds with the composition II–III_2_–VI_4_ (where II = Zn, Cd, or Hg; III = Al, Ga, or In; VI = S, Se, or Te) have been studied extensively over the globe since 1955.^18^ II–III_2_–VI_4_ group compounds are earth-abundant, less expensive, and less toxic; they are largely derived from the zinc blende structure using the Grimm–Sommerfel rule. According to Hahn *et al.*,^18^ ternary semiconducting compounds of composition II–III_2_–VI_4_ have a space group *S*^2^_4_(*I*4̄). They are thiogallates that crystallize into a tetragonal structure^19^ and are distinguished from chemical and iso-electronic analogs with the structure of chalcopyrite and sphalerite by the presence of an ordered cation vacancy. As a result, compounds of composition II–III_2_–VI_4_ are known as defective chalcopyrite (DC).

The II–III_2_–VI_4_ family's CdIn_2_Se_4_ has enthralled substantial contemplation from scientists over the orb due to its prerogatives in thermoelectric materials,^20,21^ photoanodes,^22^ heterojunction solar cells,^23^ PEC solar cells,^24–28^ photovoltaics,^29^*etc.* The structural, morphological, electrical, optical, mechanical, vibrational, and thermoelectric properties of CdIn_2_Se_4_ in bulk and/or thin film form have been explored by various authors.^30–34^ Experimental studies of the ordered-vacancy compound CdIn_2_Se_4_ have revealed that its tetragonal structure can be deformed with a lattice constant ratio *c*/*a* of 1, 2, and 3, which are referred to as the α (pseudo-cubic), β, and γ phases, respectively, depending on the synthesis conditions.^31^ Because of their high absorption coefficient, optical band gap energy (*E*_g_) in the visible spectra, and relatively high photo-electronic sensitivity from the visible to the near-infrared spectrum range, CdIn_2_Se_4_ thin films are a propitious entrant for photonic devices. As an upshot, research on their optical characteristics is of prodigious concern.

When depositing ternary semiconducting compounds with constituents that have varying vapor pressures, pulsed laser deposition (PLD) is an excellent choice. Comparing PLD technology to traditional vacuum-based thin film deposition methods, the former reduces processing times by enabling the growth of uniform and fast thin films. Of particular relevance is the ease with which heterojunctions and multilayer structures requiring precise control over film thickness can be produced using PLD technology. In the current study, the authors employed PLD to deposit CdIn_2_Se_4_ thin films because it offers several benefits over other thin film deposition methods, such as outstanding thickness controllability, broad universality, high-level cleanliness in the deposited film, and reproducible maintenance of the target material's precise composition.^35,36^

In the current study, amorphous quartz glass (fused silica) was employed to deposit CdIn_2_Se_4_ thin films for their optical characterization. Due to its excellent transparency in the UV-Visible-NIR spectra, quartz glass is perfect for examining the optical characteristics of thin films without the substrate affecting the results. Characterizing the thin film's intrinsic optical behavior is easier because quartz glass is non-crystalline and does not introduce diffraction patterns or crystallographic effects. Because it does not react with many solvents and can tolerate high processing temperatures, quartz is useful for post-treatment and film deposition procedures. The smooth surface of high-quality quartz glass usually aids in producing homogeneous films and reliable optical data.

In the extant investigation, an attempt has been made to acquire exhaustive information on the effect of substrate temperature (*T*_s_) on the optical properties of pulsed laser deposited α-phase CdIn_2_Se_4_ thin films by engaging sophisticated techniques such as ultraviolet-visible-near infrared (UV-VIS-NIR) and Fourier transform infrared (FTIR) spectrophotometers by keeping CdIn_2_Se_4_ thin film's thickness (*d*) identical throughout the investigation. The authors believe that the contemporary enquiry on the hitherto unfabricated-unpublished-unreported, *T*_s_-controlled optical characterizations of pulsed laser deposited α-phase CdIn_2_Se_4_ thin films should not only be an accumulation to the prevailing list but also lead to the further understanding of the optical properties of thin films and the research will afford optimized optical parameters for designing and developing of future novel semiconducting compound thin film opto-electronic device applications.

## 2 Experimental

For their optical characterizations, CdIn_2_Se_4_ ternary semiconducting compound thin films were deposited using the PLD technique (model: Compex-Pro Excimer Laser 102F; make: Coherent, Germany). The source (target) material was a single-phase CdIn_2_Se_4_ (ref. 37) in a pellet form with 10 mm (diameter) × 3 mm (thickness) dimensions. The substrates were ultrasonically cleaned amorphous quartz glass (make: Blue Star, made: Polar Industries Corporation, India) with dimensions 10 ± 0.1 mm (length) × 3 ± 0.1 mm (breadth) × 1.45 ± 0.1 mm (thickness). A CdIn_2_Se_4_ pellet and amorphous quartz glass were mounted in a stainless-steel PLD chamber (make: Excel Instruments, India). Silver conductive adhesive paste (model: RS pro-RS186-3600, made: RS Components & Controls Limited, India) adheres the CdIn_2_Se_4_ pellet to the target holder and amorphous quartz glass substrates to the substrate holder. The CdIn_2_Se_4_ pellet was irradiated by a krypton fluoride (KrF) pulsed laser through a quartz lens. The plasma plume dimension at the CdIn_2_Se_4_ pellet was optimized and confined using a quartz lens. 45° separates the substrate holder from the incident laser that cataracts on the target. The target was spun at six revolutions per minute (rpm) during laser ablation to avert mutilation from dropping laser stucks at a single spot incessantly on the CdIn_2_Se_4_ target.

The thin films of CdIn_2_Se_4_ were deposited on the amorphous quartz glass substrate at diverse *T*_s_, vacillating from room temperature (RT) (≃300 K) to 675 K (300 K ≤ *T*_s_ ≤ 675 K) using a calibrated chromel–alumel (Cr–Al) thermocouple abetted microcontroller-based temperature controller (make: Coherent, India). The *T*_s_ were sensed and controlled during the thin film deposition by mounting the chromel–alumel (Cr–Al) thermocouple on the quartz glass substrate surface. The thin film *d* and deposition rate was monitored and/or controlled by the *in situ* digital quartz crystal thickness monitor (model: DTM-101, make: Hind High Vacuum Co. Pvt. Ltd, India). During CdIn_2_Se_4_ thin films' deposition, the evaporation rate (≃10 nm s^−1^) and the *d* of the deposits (≃100 nm) were kept constant. Table 1 displays the PLD parameters optimized for synthesizing CdIn_2_Se_4_ thin films.

**Table 1:** PLD parameters optimized for the synthesis of CdIn_2_Se_4_ thin films

S. no.	Parameter	Value
1	KrF laser wavelength (*λ*)	≃248 nm
2	KrF laser energy	≃255 mJ
3	Repetition rate (frequency)	≃04 Hz
4	Source-to-substrate distance	≃38 mm
5	Substrate temperature (*T*_s_)	300 K ≤ *T*_s_ ≤ 675 K
6	Thickness of thin films (*d*)	≃100 nm
7	Base vacuum	≃1.00 × 10^−4^ Pa
8	Deposition time (laser ablation time)	05-to-20 minutes

The variable laser ablation duration was used to achieve consistent *d* at all *T*_s_. It was observed that the thin films deposited at lower *T*_s_ require less laser ablation time during the synthesis of thin films, whereas thin films synthesized at higher *T*_s_ require more laser ablation time to achieve the same *d* as those deposited at lower *T*_s_ because of re-evaporation at high *T*_s_.

The effect of the *T*_s_ (300 K ≤ *T*_s_ ≤ 675 K) on the optical properties of pulsed laser deposited CdIn_2_Se_4_ thin films was scrutinized by recording room temperature (RT) (≃300 K) transmittance (*T*(*λ*)) spectra employing UV-Vis-NIR spectrophotometer (model: Lambda 1050+, make: PerkinElmer, USA) in the 400 to 900 nm wavelength (*λ*) range. FTIR spectrophotometer (model: IRSpirit-X, make: Shimadzu, Japan) operated in mid-infrared mode at room temperature (RT) (≃303 K) in the wavenumber (*ν̄*) range 4000–400 cm^−1^ with a resolution of 2 cm^−1^ to spot the functional groups existing (if any) in the pulsed laser deposited CdIn_2_Se_4_ thin films deposited at various *T*_s_.

The thin film characteristics altered depending on numerous pre- and post-deposition conditions. All other PLD parameters itemized in Table 1 were held constant during the current experiments, except for *T*_s_ and deposition duration, to ensure the reproducibility of the findings.

## 3 Results and discussion

### 3.1. UV-vis-NIR spectrophotometer analysis

The UV-VIS-NIR spectrophotometer was employed to derive optical spectra of *T*_s_-modified CdIn_2_Se_4_ thin films. UV-VIS-NIR spectrophotometer can produce optical spectra in a variety of modes, including *T*(*λ*), reflectance (*R*(*λ*)), and absorption (*A*(*λ*)), depending on the materials' structure and characteristics. An opaque sample, such as pellet, bulk, *etc.*, has awfully low transparency, often imminent to zero (≃0), and the optical parameters are resolute when measured by consuming the diffuse reflectance spectra (DRS), which use the *R*(*λ*) spectra as per Kubelka–Munk (K–M) theory.^38^ According to Jubu *et al.*,^39^ the Tauc technique evaluates the optical characteristics of a transparent sample by using the absorption (*A*(*λ*)) and/or *T*(*λ*) spectra of the UV-VIS-NIR spectrophotometer.

**Fig. 1:**
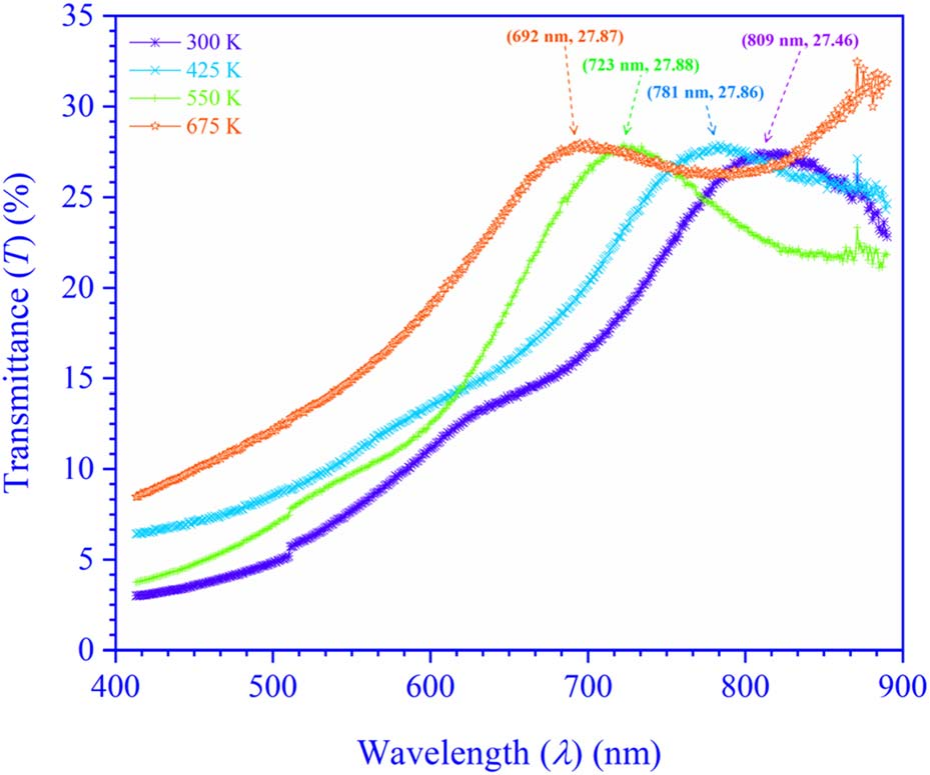
Transmittance spectra of *T*_s_-moderated CdIn_2_Se_4_ thin films.

The current study analyzes *λ*-dependent room temperature (RT) (≃300 K) *T*(*λ*) spectra, Fig. 1, for *T*_s_ tempered CdIn_2_Se_4_ thin films using a UV-VIS-NIR spectrophotometer in the *λ* range of 400 to 900 nm.

The shift in *λ* dependent *T*(*λ*) maxima perceptible in Fig. 1 can be explained by the thin films of the CdIn_2_Se_4_ formed at low *T*_s_ (*T*_s_ < 425 K) have dispersed microcrystallites, random orientation, disordered grain boundaries, and are essentially amorphous, which contributes to lower *T*(*λ*) and increased absorption (*A*(*λ*)). Conversely, CdIn_2_Se_4_ thin films grown at higher *T*_s_ (300 K < *T*_s_ < 675 K) have a stronger orientation, organized microcrystallites, starkly defined grain boundaries, and are essentially polycrystalline, all of which contribute to elevated *T*(*λ*) and diminished absorbing (*A*(*λ*)) capabilities. When deposited at 675 K *T*_s_ (>550 K), thin films of the CdIn_2_Se_4_ show an additional phase (In_2_Se_3_)^40^ that could be the cause of the *T*(*λ*) and absorption (*A*(*λ*)) divergence. The characteristic wavelengths (*λ*_c_) of *T*_s_ modified CdIn_2_Se_4_ thin films with their *T*(*λ*) amplitude are revealed in Fig. 1. The *T*_s_ modulated CdIn_2_Se_4_ thin films has high absorption in the visible region with a hump at 750.00 ± 58.50 nm making it a good choice for light absorbing material.

**Fig. 2:**
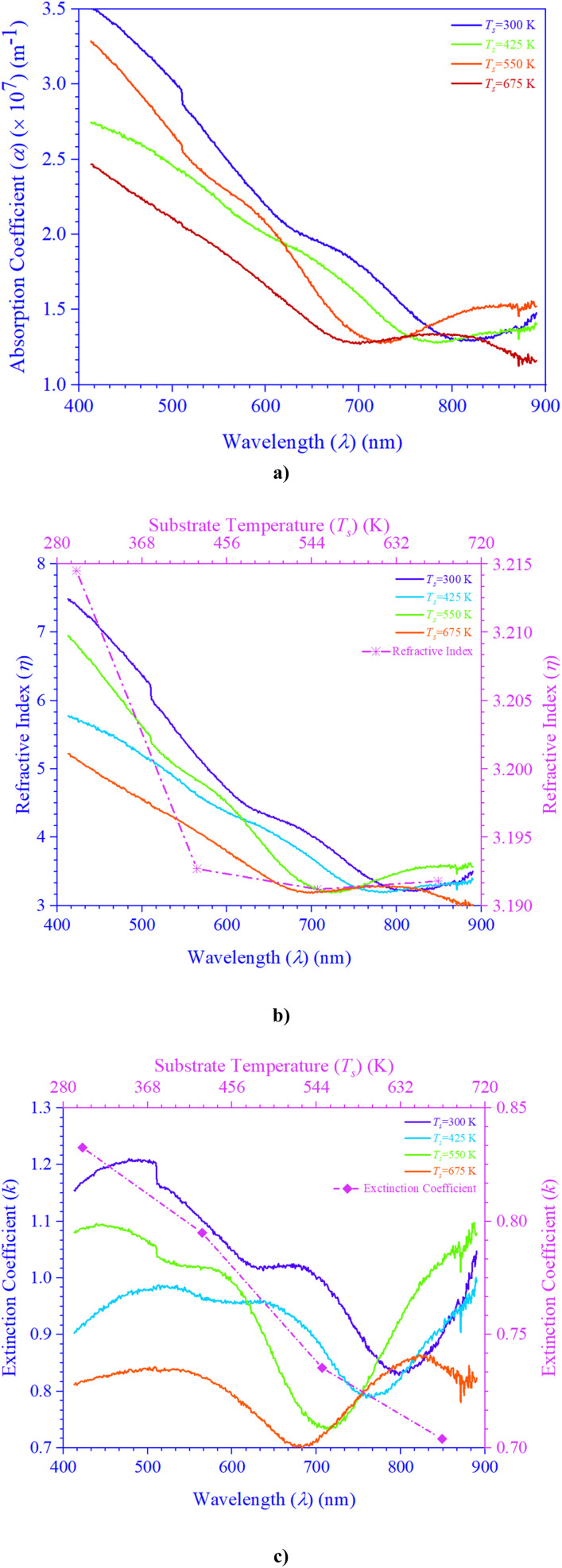
*T*_s_ modulated CdIn_2_Se_4_ thin films' (a) absorption coefficient, (b) refractive index, and (c) extinction coefficient.

Using eqn (1)–(3), Fig. 2 demonstrates how the optical parameters—the absorption coefficient (*α*), refractive index (*η*), and extinction coefficient (*k*)—vary with *λ* for *T*_s_ curbed CdIn_2_Se_4_ thin films.1
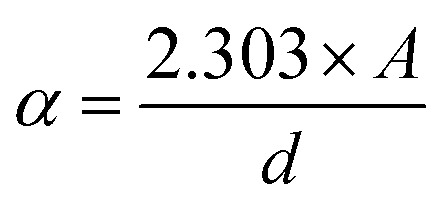
2
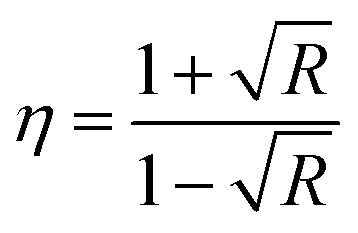
3
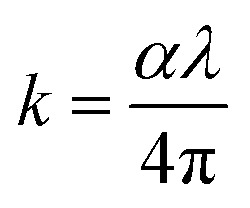


Eqn (1) shows that the *d* is ≃100 nm for CdIn_2_Se_4_ thin films.

The *α* scale depends upon radiation photon energy (*hν*) and the structure of a thin film. Bulk and/or thin films with higher *α* absorb photons more readily, which excite electrons into the conduction band (CB). The higher value of *α* (≃10^7^ m^−1^)^32,41^ of *T*_s_ curbed CdIn_2_Se_4_ thin films palpable in Fig. 2(a) supports the direct optical band gap (*E*_g_) nature of the semiconductor^32^ and makes it a very good candidate for designing and developing opto-electronic devices.^42^

Fig. 2(b) shows *R*(*λ*) dependent 
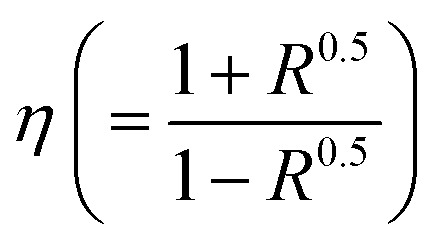
 spectra as a function of *λ* for *T*_s_-controlled CdIn_2_Se_4_ thin films. The dispersion curves of the *η* show crests at *λ*_c_, which the oscillator model can explain. As per El-Nahass *et al.*,^43^ at a *λ* higher than the *λ*_c_, the spectral behavior of the *η* parades normal dispersion, which the single oscillator model can explicate. The *η* values deduced for *T*_s_ tempered CdIn_2_Se_4_ thin films, stake well with the empirical relation *η*^4^(*E*_g_ − 0.365) = 154 (*η* ≃ 3.27) agreed by Reddy *et al*.^31,44,45^

The *k* quantifies the percentage of light energy lost through scattering and/or absorption per unit transit distance in thin films, reflecting the absorption of electromagnetic waves due to inelastic scattering events. Through empirical observation, the *k* is proportional to the 
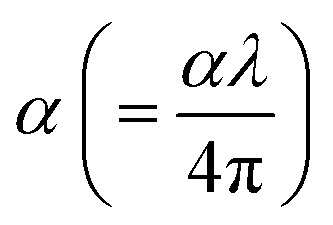
. Fig. 2(c) shows the change in *k* with *λ* for *T*_s_ modified CdIn_2_Se_4_ thin films. The *k* decreases with an increase in *λ* and reaches a minimum at *λ*_c_; the increment in *k* values after *λ*_c_ can be elucidated due to the free carrier absorption contribution; the behavior agrees well with the results reported by El-Nahass *et al*.^43,45^

The course used to synthesize the bulk and/or thin film, the thickness of the bulk and/or thin film (*d*), *T*_s_ at which thin films were/are grown, *etc.*, could all be causative factors to the variation in the *α*, *η*, and *k* values for the bulk and/or thin film CdIn_2_Se_4_.

To reach the lowest energy optically excited state, the incident photon energy (*hν*) is designated by the optical band gap (*E*_g_). The Tauc technique construes the *E*_g_ of *T*_s_, moderated CdIn_2_Se_4_ thin films. The optical *α* is correlated to the *E*_g_ for interband transitions in a semiconductor, adjacent to the *E*_g_ by engaging eqn (4).^46^4
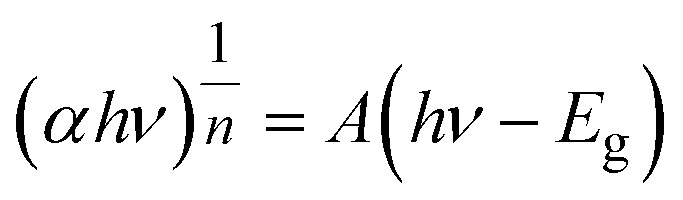
In eqn (4), the transition probability is symbolized by *n* (=2, 1/2, 2/3, and 1/3 for direct allowed, indirect allowed, direct forbidden, and indirect forbidden transitions, respectively), and *A* is a constant that is contingent on the nature of the transition.

**Fig. 3:**
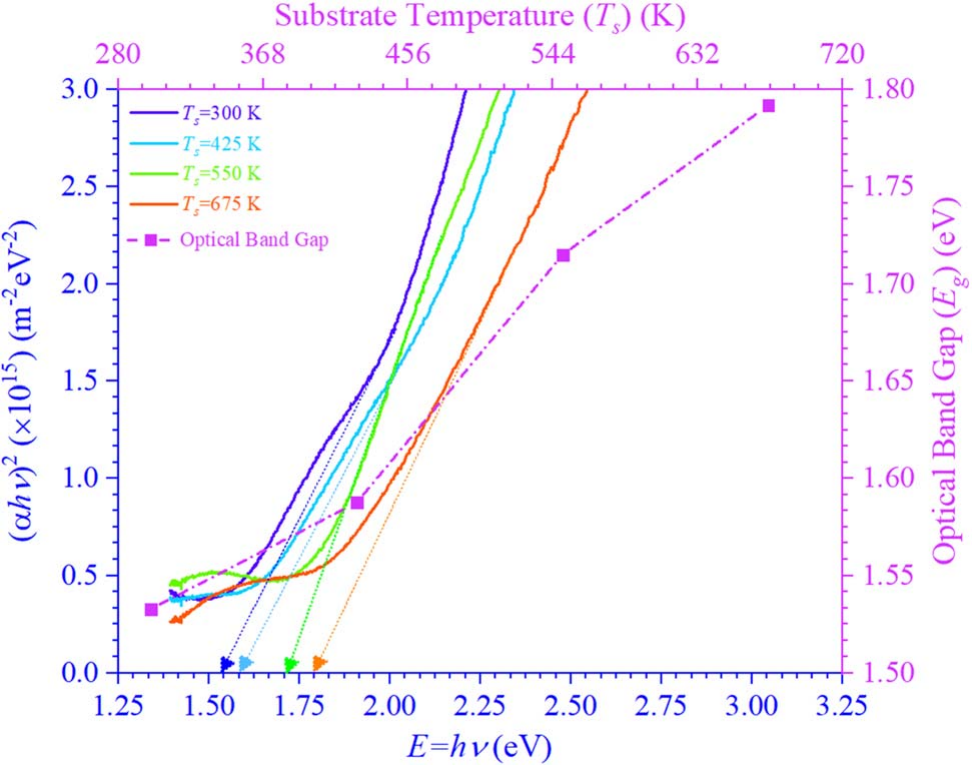
Dependency of (*αhν*)^2^ on the photon energy for *T*_s_-controlled CdIn_2_Se_4_ thin films.

Photons have less energy than the band gap; therefore, materials in any form—bulk or thin film—cannot absorb light beneath it. Fig. 3 shows the plot of (*αhν*)^2^*vis-à-vis* photon energy (*hν*), which is used to find the *E*_g_ values of *T*_s_ curbed CdIn_2_Se_4_ thin films. The direct *E*_g_ values may be articulated by extrapolating the straight segment of the graphs at the values where (*αhν*)^2^ tends to zero (≃0).

The *E*_g_ values of the thin films grown below 550 K *T*_s_ (<550 K) *i.e.* at 300 K and 425 K are found to be ≃1.53 eV and ≃1.59 eV, which are slightly less than the CdIn_2_Se_4_'s reported direct *E*_g_ value ≃1.67 eV attainable for pure α-phase CdIn_2_Se_4_ crystal derived by optical absorption method at 293 K temperature.^19^ Nevertheless, CdIn_2_Se_4_ thin films grown at 550 K *T*_s_ (≃550 K) have an *E*_g_ value (≃1.72 eV) which is more or less equal to the reported bulk value. At higher *T*_s_ (>550 K), the higher value of the *E*_g_ (≃1.79 eV) can be explained by the dissociation of CdIn_2_Se_4_ thin film.^40^ The *E*_g_ value increases with the *T*_s_ increase. As the *T*_s_ of CdIn_2_Se_4_ thin films rose, as ostensible in Fig. 1, *T*(*λ*) changed toward higher energy (=*hν*), signifying that the optical band gap energy widened concurrently. The increase in *E*_g_ with increase in *T*_s_ may be explained due to the higher *T*_s_ providing atoms greater energy to rearrange into well-ordered crystal structures, they diminish grain boundaries and dislocations, which typically bring localized energy states within the *E*_g_. Larger grain sizes in polycrystalline films frequently result from higher *T*_s_; dominating grain growth improves electronic properties and widens the *E*_g_.^47–51^ The *E*_g_ value construed in the present investigation advocates that 550 K *T*_s_ deposited CdIn_2_Se_4_ thin films are hypothetically adequate for thin film solar cell applications.^52^

There is diversity in the literature about the type of electronic transition and the corresponding *E*_g_ values. The *E*_g_ values of *T*_s_ tempered CdIn_2_Se_4_ thin films determined in the present investigation differ when compared with the literature survey;^23,24,45,52,53^ the incongruities explicated may be due to the individual or combined consequence of some factors like incongruent nature of the melting of CdIn_2_Se_4_, innumerable methods employed for synthesizing the compound and/or thin film deposition, *T*_s_, an aberration in bulk and/or thin film's stoichiometry, charge impurities at the grain boundaries, lattice strain present in bulk and/or thin film, extent of structural disorder, *etc.*

**Fig. 4:**
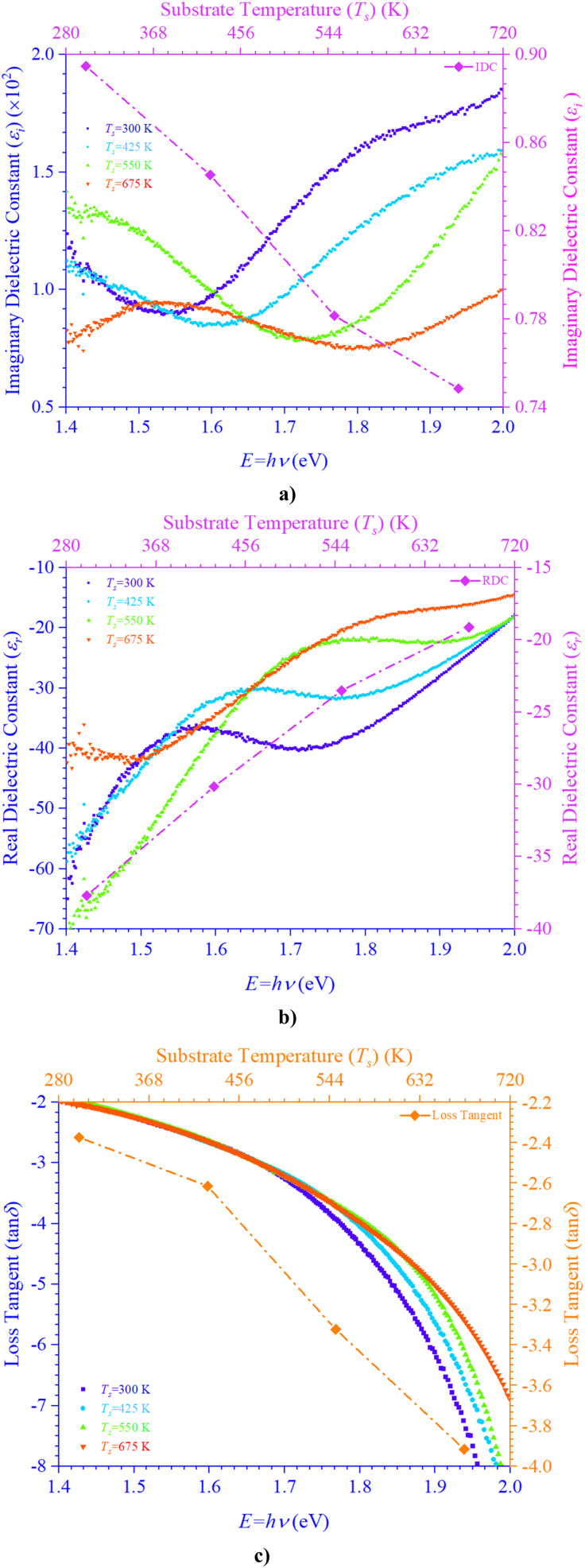
Variation in (a) IDC, (b) RDC, and (c) tan *δ* of CdIn_2_Se_4_ thin films as a function of energy.

The imaginary (IDC) (*ε*_i_) and the real (RDC) (*ε*_r_) dielectric constants are portions of the complex optical dielectric function; the IDC epitomizes the absorption of energy (*E*) from an electric field (*F*_e_), which may be pronounced due to the motion of a dipole, while the RDC embodies the capacity of materials to lessen the speed of light (*c*); IDC and RDC have unswerving kin with the *η* and *k*. Fig. 4 displays the relationship between energy and the changes in the IDC (=2*ηk*) (Fig. 4(a)), RDC (=*η*^2^ − *k*^2^) (Fig. 4(b)), and loss factor/dissipation factor/loss tangent 
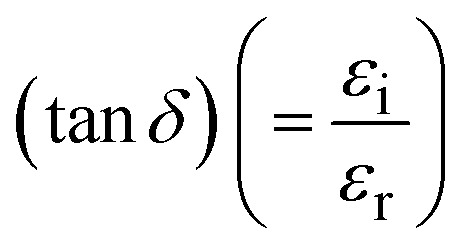
 (Fig. 4(c))) for *T*_s_ modified CdIn_2_Se_4_ thin films. It is evident from Fig. 4 that IDC varies from 0.89 × 10^2^ to 0.75 × 10^2^, RDC from −37.69 to 19.12, and tan *δ* from −2.37 to −3.92 with a change in *T*_s_.

The negative values of the real dielectric constant (*ε*_r_) indicate that the semiconductor materials reveal metallic behavior.^54^

**Fig. 5:**
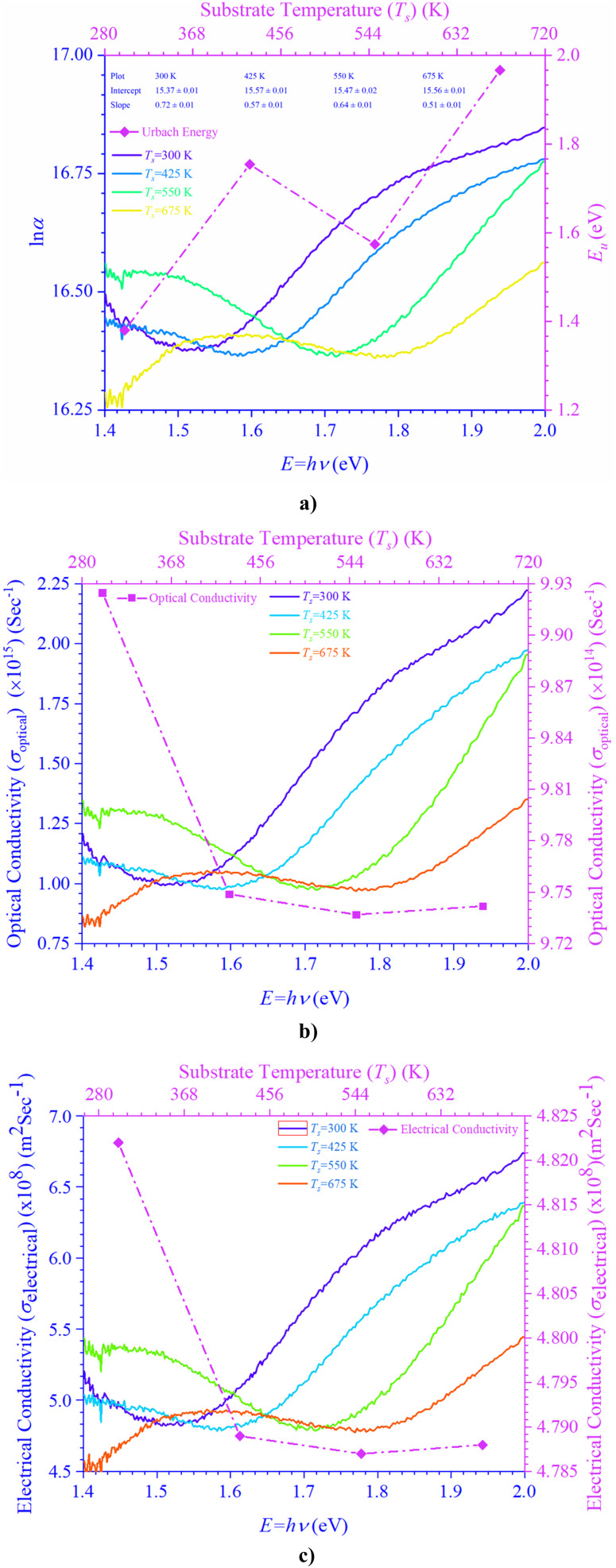
Variation in (a) Urbach's energy, (b) optical conductivity, and (c) electrical conductivity of CdIn_2_Se_4_ thin films as a function of energy.

The rapport between *E*_g_ (=*hν*) and the vicissitudes in the natural logarithm of *α* (ln *α*), optical conductivity (*σ*_o_), and electrical conductivity (*σ*_e_) is revealed in Fig. 5. An alteration in optical state occurs when the valence band (VB) tail becomes occupied, and the conduction band (CB) edge becomes unoccupied, as revealed by eqn (5).^55^5
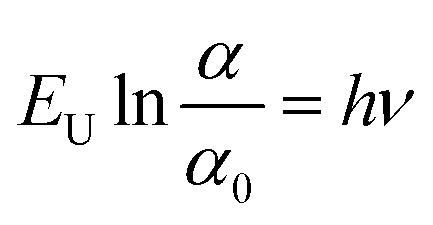
In this context, *α*_0_ represents an Urbach absorption coefficient (constant), and *E*_U_, which stands for Urbach's energy, determines the slope of the exponential edge and can be seen as the width of the tail of localized states in the forbidden energy gap. The thermal vibrations of the lattice form the basis of the *E*_U_. The values of the *E*_U_ and *α*_0_ attained from the ln *α* against photon energy (*hν*) plot varies from 1.38 to 1.97 eV and 4.75 × 10^6^ to 5.73 × 10^6^ m^−1^, respectively, with a change in *T*_s_.

The high density of localized states inside the *E*_g_, as implied by the enormous *E*_U_ value, reveals numerous structural flaws in sample.^55^ The powerful probes, 
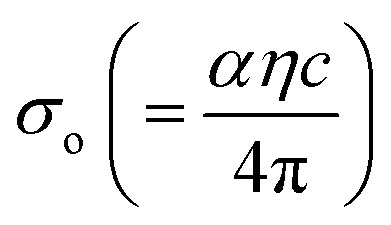
 and 
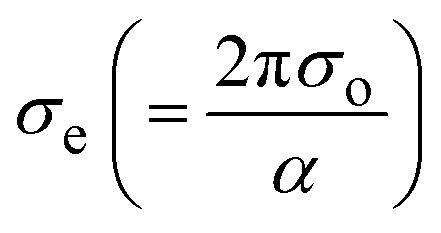
 represent the mobility of the charge carriers induced by the alternating electric field of the passing electromagnetic waves,^55^ and are employed in investigating the electrical properties of different materials. The values of *σ*_0_ and *σ*_e_ for *T*_s_ curbed CdIn_2_Se_4_ thin films extracted at *λ*_c_ are plotted in Fig. 5(b) and (c), respectively.

The *σ*_e_ is reliant on *σ*_o_, while *σ*_o_ is based on the *α*. As light absorption grows, *σ*_o_ and *σ*_e_ climb, enabling electrons to acquire more energy and become free for conduction.^56^

**Fig. 6:**
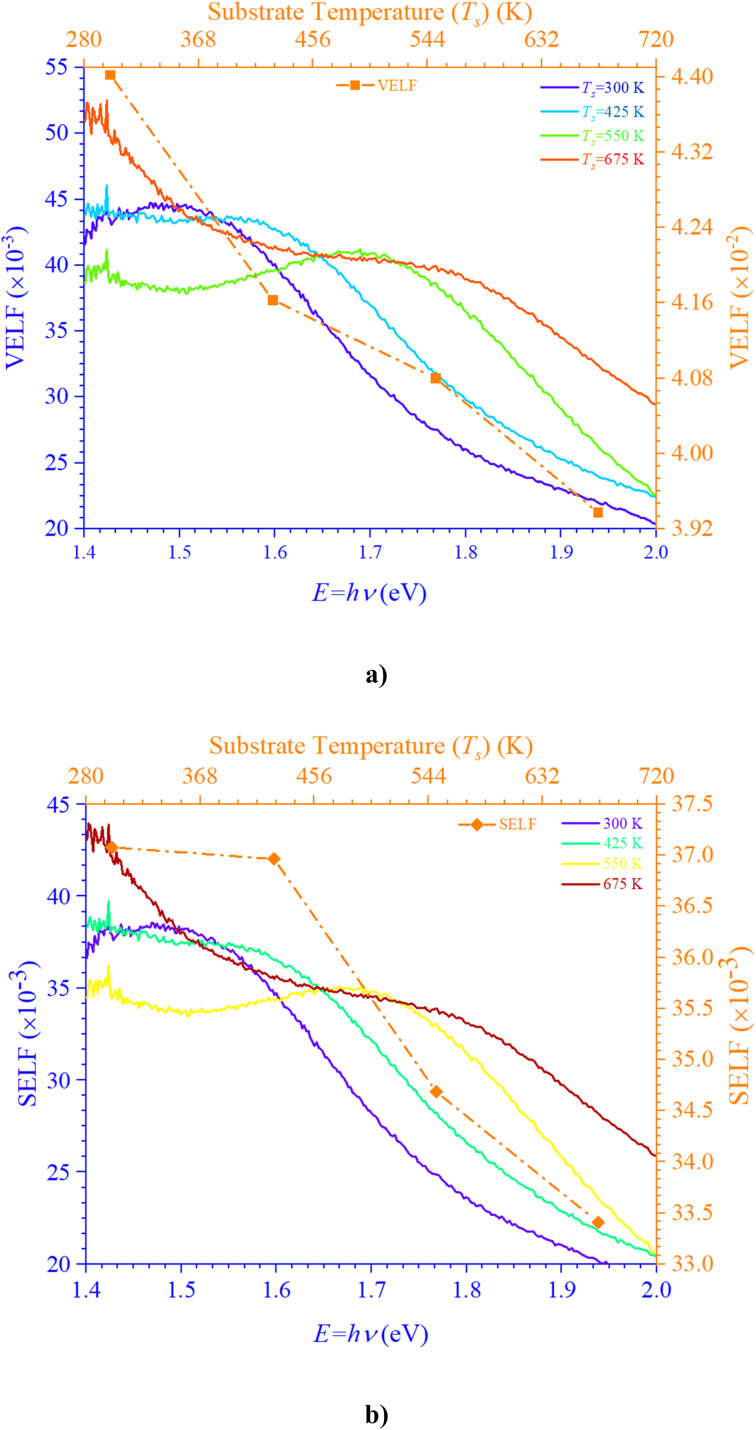
Variation in (a) VELF and (b) SELF of CdIn_2_Se_4_ thin films as a function of energy.

Fig. 6 portrays IDC and RDC reliant volume 
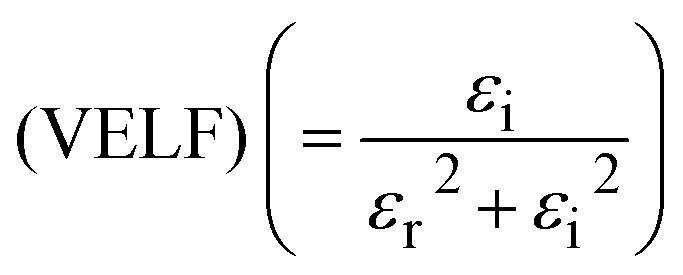
 (Fig. 6(a)) and surface 
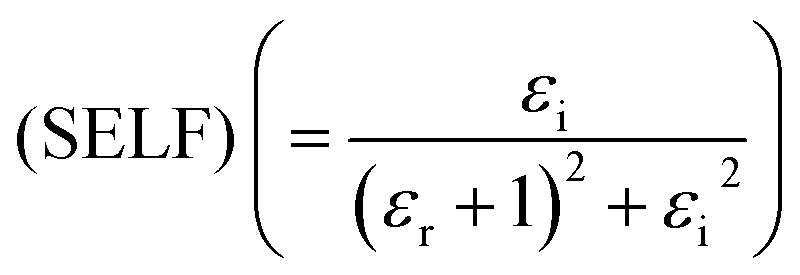
 (Fig. 6(b)) energy loss functions depict electron and optical transitions in *T*_s_, moderated CdIn_2_Se_4_ thin films. The VELF and SELF are used to weigh the energy loss rates of electrons as they move across most of the surface. The peak values of VELF and SELF for *T*_s_ curbed CdIn_2_Se_4_ thin films extracted at *λ*_c_ vary from 44.03 × 10^−3^ to 39.37 × 10^−3^ and 37.08 × 10^−3^ to 33.41 × 10^−3^, respectively.

### 3.2. FTIR spectrophotometer analysis

**Fig. 7:**
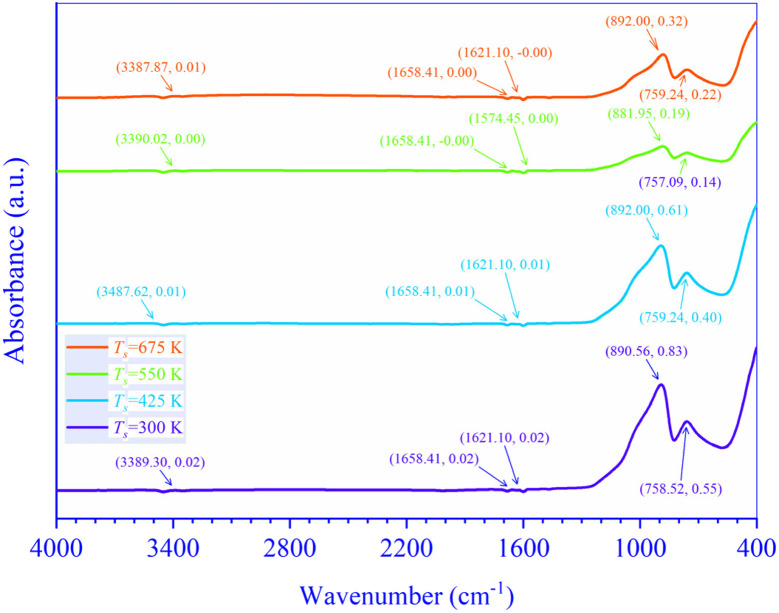
FTIR spectra of *T*_s_ modified CdIn_2_Se_4_ thin films.

The room temperature (RT) (≃300 K) FTIR spectra of *T*_s_ controlled CdIn_2_Se_4_ thin films recorded in the 4000 to 400 cm^−1^ wavenumber (*ν̄*) range is offered in Fig. 7.

In synthesizing CdIn_2_Se_4_, only 5N (99.999%) pure Cd, In, and Se were employed; no other chemicals or chemical routes were used, and a non-chemical approach was used to deposit CdIn_2_Se_4_ thin films.

A peak perceived in *T*_s_ tempered CdIn_2_Se_4_ thin films between 880–892 cm^−1^ wavenumber (*ν̄*) can be construed due to bending C=C and/or C−H functional groups and alkene and/or 1,2,4-trisubstituted and/or 1,3-disubstituted class. A peak seized between 757 and 760 cm^−1^ wavenumber (*ν̄*) can be interpreted due to stretching C−Cl and halo compound functional group, and/or bending C=H functional group and 1,2-disubstituted or monosubstituted class. The absorption peaks due to C=C, C−H, and/or C−Cl functional group/s habitually parade a stout absorption edge in the FTIR spectra if existing in the *T*_s_ modified CdIn_2_Se_4_ thin films. The absorption edges are evident in the *T*_s_ controlled CdIn_2_Se_4_ thin films' FTIR spectra between 880–892 cm^−1^ and 757–760 cm^−1^ wavenumbers (*ν̄*), but they are frail; henceforth, it may be concluded that the *T*_s_ moderated CdIn_2_Se_4_ thin films are intrinsic and free from any functional group.^21,57^ The shift in wavenumber (*ν̄*) dependent absorbance (*A*) maxima conspicuous in Fig. 7 can be explained by the amorphous, polycrystalline, and polyphase nature of the *T*_s_ controlled CdIn_2_Se_4_ thin films. Peaks in FTIR spectra of *T*_s_ tempered CdIn_2_Se_4_ thin films at wavenumbers (*ν̄*) 3437.74 ± 49.87 cm^−1^, 1658.41 cm^−1^, and 1597.77 ± 23.32 cm^−1^ are detected and dispensed by the instrument, but their amplitude is impending almost to zero (≃0), so they are not identified and indexed.

## 4 Conclusions

The optical characterization of the PLD technique deposited ≃100 nm thick CdIn_2_Se_4_ thin films on amorphous quartz glass at mixt *T*_s_ was carried out by recording RT *T*(*λ*) spectra in the 400 to 900 nm *λ* range using a UV-Vis-NIR spectrophotometer. A shift in *T*_s_ modulated CdIn_2_Se_4_ thin film's *T*(*λ*) maxima is evident and exhibits high absorption in the visible region with a hump at 750.00 ± 58.50 nm and depicts *α* of the order of ≃10^7^ m^−1^. The *η* values deduced for *T*_s_ tempered CdIn_2_Se_4_ thin films (≃3.27). The *k* decreases with an increase in *λ* and reaches a minimum at *λ*_c_. Because of their amorphous nature, the *E*_g_ of CdIn_2_Se_4_ thin films grown below 550 K *T*_s_ is slightly lower than the published value; while the polycrystalline thin films of CdIn_2_Se_4_ made at a *T*_s_ of 550 K have an *E*_g_ value that is approximately equal to the reported bulk value, the polyphase structure of the CdIn_2_Se_4_ thin film at higher *T*_s_ explains greater values of the *E*_g_. For *T*_s_-modulated CdIn_2_Se_4_ thin films, IDC, RDC, and tan *δ*, dependent on *η* and *k* have all been determined and compared with the reported data. The values of the *E*_U_, *α*_0_, *σ*_o_, *σ*_e_, VELF, and SELF of CdIn_2_Se_4_ thin films limited by *T*_s_ were retrieved at *λ*_c_. The purity of CdIn_2_Se_4_ thin films formed at different *T*_s_ was verified by recording the FTIR spectra. The conclusions drawn from our extensive research include that the *T*_s_-dependent optical parameters obtained in this study will aid in developing upcoming thin film electronic devices in which CdIn_2_Se_4_ thin film is one of the semiconducting materials. The impact of thin film *d* on the optical characteristics of CdIn_2_Se_4_ thin films deposited by PLD has not been investigated by researchers worldwide, despite the conclusion that this is a topic of future research. Research on compounds based on the II–III_2_–VI_4_ group in silicon-based microelectronic devices is underway. However, no attempt has been made to create a junction between silicon and CdIn_2_Se_4_, so the results of this study may provide insight into the development of new silicon-based microelectronic devices in the future. Additionally, the current research suggests that CdIn_2_Se_4_ thin films are potential choices for designing and developing future high-efficiency opto-electronic systems.

## Author contributions

All writers contributed to the study conception and design. Material preparation, data gathering, and analysis were accomplished by S. D. Dhruv, Tanvi Dudharejiya, Sergei A. Sharko, Aleksandra I. Serokurova, Nikolai N. Novitskii, D. L. Goroshko, Parth Rayani, Jagruti Jangale, Vanaraj Solanki, P. B. Patel, U. B. Trivedi, J. H. Markna, Bharat Kataria, and D. K. Dhruv. D. K. Dhruv wrote the first draft of the manuscript, and all authors commented on previous versions. All authors read and permitted the final manuscript. S. D. Dhruv: methodology. Tanvi Dudharejiya: conceptualization. Sergei A. Sharko: investigation. Aleksandra I. Serokurova: formal analysis. Nikolai N. Novitskii: *visualization*. D. L. Goroshko: writing-review & editing. Parth Rayani: software. Jagruti Jangale: validation. Vanaraj Solanki: data curation. P. B. Patel: resources. U. B. Trivedi: resources. J. H. Markna: software. Bharat Kataria: project administration. D. K. Dhruv: writing-original draft, supervision.

## Conflicts of interest

The submitted work is the authors' original research work and has not been communicated elsewhere for publication. On behalf of all co-authors, I declare no conflict of interest.

## Data Availability

The authors declare that the data supporting the findings of this study are available/included in the paper's main text.
